# Evaluation of Timing and Route of Epinephrine in a Neonatal Model of Asphyxial Arrest

**DOI:** 10.1161/JAHA.116.004402

**Published:** 2017-02-18

**Authors:** Payam Vali, Praveen Chandrasekharan, Munmun Rawat, Sylvia Gugino, Carmon Koenigsknecht, Justin Helman, William J. Jusko, Bobby Mathew, Sara Berkelhamer, Jayasree Nair, Myra H. Wyckoff, Satyan Lakshminrusimha

**Affiliations:** ^1^ Pediatrics UC Davis Sacramento CA; ^2^ Pediatrics SUNY University at Buffalo NY; ^3^ Pharmaceutical Sciences SUNY University at Buffalo NY; ^4^ Pediatrics UT Southwestern Dallas TX

**Keywords:** cardiac arrest, cardiac arrhythmia, epinephrine, neonate, pharmacokinetics, resuscitation, Cardiopulmonary Arrest, Cardiopulmonary Resuscitation and Emergency Cardiac Care, Arrhythmias, Animal Models of Human Disease

## Abstract

**Background:**

Epinephrine administered by low umbilical venous catheter (UVC) or endotracheal tube (ETT) is indicated in neonates who fail to respond to positive pressure ventilation and chest compressions at birth. Pharmacokinetics of ETT epinephrine via fluid‐filled lungs or UVC epinephrine in the presence of fetal shunts is unknown. We hypothesized that epinephrine administered by ETT or low UVC results in plasma epinephrine concentrations and rates of return of spontaneous circulation (ROSC) similar to right atrial (RA) epinephrine.

**Methods and Results:**

Forty‐four lambs were randomized into the following groups: RA epinephrine (0.03 mg/kg), low UVC epinephrine (0.03 mg/kg), postcompression ETT epinephrine (0.1 mg/kg), and precompression ETT epinephrine (0.1 mg/kg). Asystole was induced by umbilical cord occlusion. Resuscitation was initiated following 5 minutes of asystole. Thirty‐eight of 44 lambs achieved ROSC (10/11, 9/11, and 12/22 in the RA, UVC, and ETT groups, respectively; subsequent RA epinephrine resulted in a total ROSC of 19/22 in the ETT groups). Median time (interquartile range) to achieve ROSC was significantly longer in the ETT group (including those that received RA epinephrine) compared to the intravenous group (4.5 [2.9–7.4] versus 2 [1.9–3] minutes; *P*=0.02). RA and low UVC epinephrine administration achieved comparable peak plasma epinephrine concentrations (470±250 versus 450±190 ng/mL) by 1 minute compared to ETT values of 130±60 ng/mL at 5 minutes; *P*=0.03. Following ROSC with ETT epinephrine alone, there was a delayed peak epinephrine concentration (652±240 ng/mL).

**Conclusions:**

The absorption of ETT epinephrine is low and delayed at birth. RA and low UVC epinephrine rapidly achieve high plasma concentrations resulting in ROSC.

## Introduction

Birth asphyxia accounts for about 23% of the ≈4 million neonatal deaths that occur each year worldwide.^1^ The majority of newborn infants require little assistance to stabilize at birth and adapt seamlessly to extrauterine life. However, ≈10% of infants require some assistance to begin breathing at birth, although less than 1% need extensive resuscitative measures such as chest compressions and epinephrine.^2^, ^3^ Beyond high rates of associated mortality, newborns who fail to respond to optimized ventilation, and who require chest compressions and pharmacologic therapy to achieve return of spontaneous circulation (ROSC), are at high risk of associated long‐term neurologic deficits.^4^, ^5^

The optimal route, order, and timing of epinephrine administration remain controversial. Most recommendations for epinephrine use are extrapolated from 1‐ to 4‐day‐old animal models that have transitioned from the fetal circulation (ie, they have decreased pulmonary vascular resistance, a closed ductus arteriosus, and possible closed ductus venosus) and have also cleared their lung fluid.^6^, ^7^ The effect and pharmacokinetics of epinephrine through an endotracheal tube (ETT) into fluid‐filled lungs, or into a low umbilical venous catheter (UVC) in the presence of fetal shunts (ductus venosus and foramen ovale), in perinatal asphyxia‐induced arrest are not known. In the clinical setting, administration of intravenous (IV) epinephrine requires placement of an UVC, so, in most instances, the first dose of epinephrine is given by the ETT route.^2^, ^8^

The current edition of the Neonatal Resuscitation Program textbook recommends administering epinephrine at a dose of 0.01 to 0.03 mg/kg through a low UVC followed by a flush of 0.5 to 1 mL of normal saline.^9^ While attempting placement of a UVC, a dose of endotracheal epinephrine may be administered at a dose of 0.05 to 0.1 mg/kg.^9^ We elected to determine the pharmacokinetics and safety profile of the upper end of these dose ranges. We hypothesized that in the setting of perinatal asphyxial arrest, high‐dose ETT epinephrine (0.1 mg/kg) delivery into fluid‐filled lungs or epinephrine administered into a low‐lying UVC (0.03 mg/kg), in the presence of a ductus venosus, will result in epinephrine concentrations and ROSC rates similar to epinephrine administered into the right atrium (0.03 mg/kg). We chose the upper limit of the recommended dose for endotracheal and umbilical venous epinephrine to optimize ROSC rates. We also hypothesized that precompression ETT epinephrine administration, prior to the initiation of chest compressions, rapidly increases subsequent plasma epinephrine concentrations and diastolic blood pressure, with the onset of chest compressions achieving quicker ROSC. We speculated that instilling epinephrine into the ETT prior to initiating positive pressure ventilation (PPV) could distribute the drug within the fluid‐filled lungs more homogeneously during the 30 s of ventilation, and then enhance/hasten the effects of epinephrine once chest compressions are started.

## Methods

### Animal Preparation

**Figure 1:**
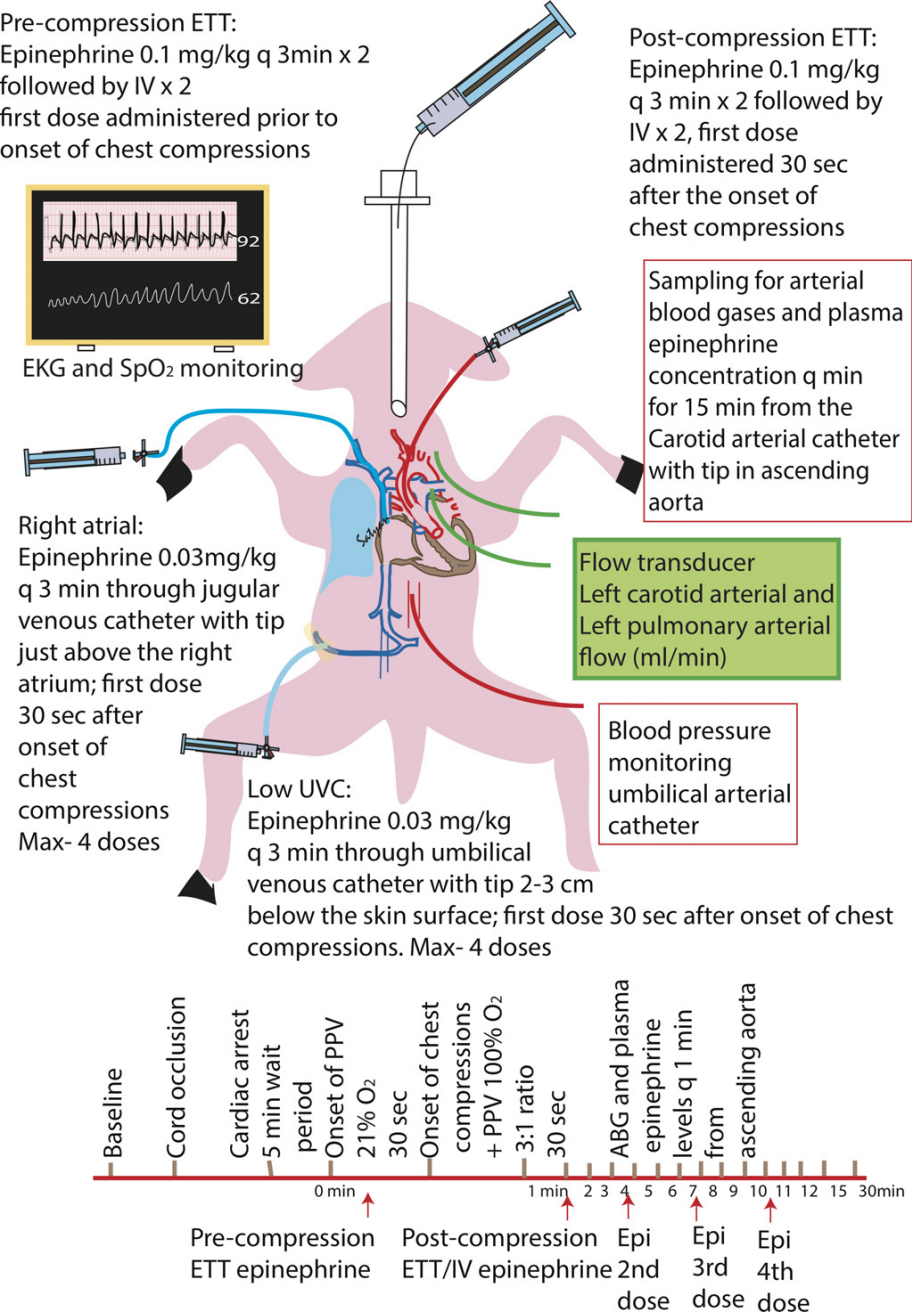
Schematic of study lamb illustrating invasive and noninvasive monitoring. ABG indicates arterial blood gas; ETT, endotracheal tube; PPV, positive pressure ventilation; UVC, umbilical venous catheter.

This study was approved by the Institutional Animal Care and Use Committee at the State University of New York at Buffalo. Time‐dated term (139–141‐day gestation) pregnant ewes were obtained from May Family Enterprises (Buffalo Mills, PA). Following an overnight fast, the ewes were induced for anesthesia with intravenous diazepam and ketamine. They were intubated with a 10.0‐mm cuffed ETT and ventilated with 21% oxygen and 2% to 3% isoflurane at 16 breaths/min. The ewes were continuously monitored with a pulse oximeter and an end‐tidal CO_2_ monitor. Following cesarean section, fetal lambs were partially exteriorized and intubated. The excess fetal lung fluid in the ETT was drained passively by gravity by tilting the head to the side to simulate loss of lung liquid with labor and, thereafter, the ETT was occluded to prevent gas exchange during gasping in the asphyxial period.^10^ Catheters were inserted into the jugular vein (for fluid and medication administration) and right carotid artery (for blood sampling). A 2‐mm flow probe (Transonic Systems Inc, Ithaca, NY) was placed around the left carotid artery. A left thoracotomy was performed and a 4‐mm flow probe was placed around the left pulmonary artery. The thoracotomy was closed in layers. ECG leads were attached at the right axilla, left axilla, and right inguinal area (3‐lead ECG). The ECG100C (Biopac Systems, Inc.) was used with Acknowledge Software to record tracings of leads I, II, and III. Preductal arterial oxyhemoglobin saturation was monitored with a pulse oximeter placed on the right forelimb of the lamb (Masimo, Irvine, CA). Following instrumentation, the umbilical cord was occluded and cut, and the lambs were moved from the maternal abdomen to the radiant warmer. During the asphyxial period, an umbilical arterial catheter was inserted in all lambs to measure continuous invasive blood pressures. A low umbilical venous catheter was inserted 2 cm below the skin (and secured after confirming blood drawing back into the catheter) in lambs randomized to receive UVC epinephrine (Figure 1).

### Experimental Protocol

Forty‐four lambs were randomized into 4 groups using opaque sealed envelopes. A 5‐minute period of asystole was observed prior to initiating resuscitation. Asystole was defined by the absence of carotid blood flow, arterial blood pressure, and heart rate (by auscultation and ECG). Resuscitation began by removing the ETT occluder and providing PPV with 21% oxygen by means of a T‐piece resuscitator at pressures of 35/5 cm H_2_O at a rate of 40 breaths/min.^10^ Pressures of 35/5 cm H_2_O are required to deliver standard tidal volumes of ≈8 to 9 mL/kg in term lambs, which is necessary to achieve normal PaCO_2_ concentrations.^11^, ^12^ Following 30 s of ventilation, chest compressions at a compression‐to‐ventilation ratio of 3:1 were commenced with a simultaneous increase in inspired oxygen to 100%. Following intravenous epinephrine administration, a flush of normal saline was given (0.5 mL/kg to ≈2 mL for a 4‐kg lamb). ETT epinephrine was administered into the hub connecting the ETT and positive‐pressure breaths were provided to help distribute the drug in the lungs as is currently recommended by the Neonatal Resuscitation Program. The concentration of IV and ETT epinephrine used was 1:10 000 (0.1 mg/mL, eg, 0.1 mg/kg=1 mL/kg). The first arterial blood sample or “arrest gas” was obtained 1 minute prior to resuscitation (4 minutes into asystole). Thereafter, blood sampling was obtained approximately every minute during resuscitation. In lambs that achieved ROSC, a blood gas sample was obtained at the time of ROSC and then every 1 to 3 minutes until 10 minutes following ROSC. Arterial blood samples were analyzed using a radiometer blood gas analyzer (ABL 800 FLEX, Denmark). Plasma epinephrine was measured at baseline, prior to cord occlusion, and at arrest. During resuscitation, epinephrine plasma concentrations were drawn prior to injection of the drug and, thereafter, every minute following epinephrine administration. Plasma epinephrine concentrations were analyzed by ELISA (Eagle Biosciences, Nashua, NY).

The lambs were randomized as follows:

*Right atrial epinephrine:* Epinephrine 0.03 mg/kg doses were administered through a jugular venous catheter advanced into the right atrium (RA) 30 s after the onset of chest compressions. If no ROSC was achieved, repeat epinephrine doses were given every 3 minutes until ROSC or for a total of 4 doses.

*Low UVC epinephrine:* Epinephrine 0.03 mg/kg was administered into the umbilical vein through a low‐lying UVC. The first dose was administered 30 s following chest compression initiation. Repeat epinephrine doses where given every 3 minutes via a low‐lying UVC.

*Postcompression ETT:* The first dose of epinephrine at 0.1 mg/kg was given 30 s after the onset of chest compressions. If ROSC was not achieved, a second dose of ETT epinephrine was given 3 minutes later. If the lambs failed to respond to ETT epinephrine, intravenous epinephrine at 0.03 mg/kg through the RA was given every 3 minutes for 2 more doses as needed.

*Precompression ETT:* The first dose of 0.1 mg/kg ETT epinephrine was administered with PPV *prior* to the onset of chest compressions. Additional doses were administered as described with postcompression ETT above.

### Data Analysis

Arterial blood flow and pressures were continuously recorded using a computer with AcqKnowledge Acquisition & Analysis Software (BIOPAC systems, Goleta, CA). Continuous variables are expressed as mean and SD. Categorical variables were analyzed using χ^2^ test with Fisher's exact test as required. Continuous variables were analyzed by 1‐way ANOVA between groups with Fisher's post hoc test within groups. Cox proportional hazards model was used to analyze time variables. SPSS 24 (IBM, Armonk, NY) was used for statistical analysis. Statistical significance was defined as *P*<0.05.

## Results

Characteristics of the lambs, including baseline hemodynamics, hemoglobin, sex distribution, birth weight, arterial blood gases, and the amount of lung liquid drained by gravity were not statistically significant among the study groups (Table 1). Cord occlusion caused severe acidosis and bradycardia in all lambs as we have previously described.^11^ Time to cardiac arrest (asystole) was comparable in the groups. The time to ROSC was significantly delayed in the ETT epinephrine groups as compared to the intravenous groups (Table 1).

**Table 1:** Baseline Characteristics, Hemodynamics, Arterial Blood Gas Analysis, and Timing and Incidence of ROSC in the 4 Study Groups

	Group 1 RA (n=11)	Group 2 UVC (n=11)	Group 3 Postcompression ETT (n=11)	Group 4 Precompression ETT (n=11)
Weight, kg	3.6±0.6	3.6±0.8	3.7±0.9	3.9±0.9
Sex (M:F)	7:4	5:6	6:5	5:6
Total lung fluid drained, mL/kg	13±3.5	15±2.6	15±6.7	18±3.8
Hemoglobin, g/dL	12.8±2.3	12±1.7	12.5±1.0	12.4±0.8
Baseline hemodynamics				
Heart rate, bpm	154±24	174±29	157±26	160±25
SBP mean, mm Hg	56±8.5	59±10	53±12	48±15
DBP mean, mm Hg	39±6.8	44±9.5	38±7.3	32±11
Mean left carotid flow, mL/kg per minute	26±7.1	32±8.5	25±8.7	27±8.8
Baseline ABG				
pH	7.22±0.11	7.10±0.09	7.15±0.08	7.16±0.14
PaCO_2_, mm Hg	64±15	67±7	69±11	68±7
PaO_2_, mm Hg	24±2.8	19±5.2	21±10	20±3.5
SpO_2_, %	64±7	54±12	61±20	57±8
Lactate, mmol/L	3.9±1.4	4.9±3.6	6.1±3.2	4.8±3.5
Time to asystole, minute	18 (16–21)	14 (12–20)	15 (14–16)	14 (12–14)
ROSC	10/11 (91%)	9/11 (82%)	8/11 (73%) After RA epi (9/11)	4/11 (36%) After RA epi (10/11)
Time to ROSC, minute	2 (1.6–2.9)	2.5 (2–3)	4 (2.9–4.8)^a^	7 (3–8)^b^

Data are mean±SD; time variables are median (interquartile ranges). ABG indicates arterial blood gas; bpm, beats per minute; DBP, diastolic blood pressure; epi, epinephrine; ETT, endotracheal tube; RA, right atrium; ROSC, return of spontaneous circulation; SBP, systolic blood pressure; SpO_2_, preductal arterial oxyhemoglobin saturation; UVC, umbilical venous catheter.

a *P*<0.05 compared to group 1.

b *P*<0.05 compared to group 1 and group 2.

### Incidence of ROSC and Tachyarrhythmias

RA epinephrine: Direct administration of epinephrine into the RA through the jugular venous catheter resulted in ROSC in 10/11 lambs. Nine lambs were resuscitated with 1 dose of epinephrine and 1 lamb required 2 doses.

UVC epinephrine: Administration of epinephrine through the low UVC resulted in ROSC in 9/11 lambs. Eight lambs received 1 dose of epinephrine and 1 lamb required 4 doses of epinephrine to achieve ROSC. One lamb developed a supraventricular tachyarrhythmia a few minutes following ROSC and could not be converted to normal rhythm despite vagal stimulation and repeated doses of adenosine (ROSC was not sustained).

Postcompression ETT epinephrine: The first postcompression dose of ETT epinephrine led to ROSC in 4/11 lambs. Four more lambs achieved ROSC following the second dose of ETT epinephrine. One lamb required 1 dose of RA epinephrine following the 2 doses of ETT epinephrine to be successfully resuscitated. Of the other 2 lambs that received IV epinephrine that did not achieve ROSC, 1 developed a tachyarrhythmia ≈2 minutes following ROSC and could not be converted into sinus rhythm despite medical treatment (ROSC was not sustained).

**Figure 2:**
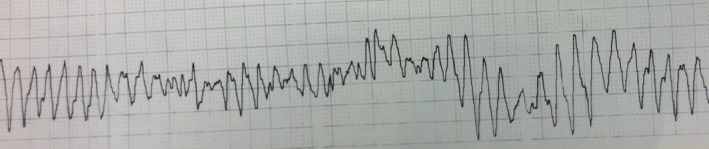
Tachyarrhythmia (torsades de pointes) in a lamb that received multiple epinephrine doses.

Precompression ETT epinephrine: Precompression ETT administration of epinephrine led to ROSC in 4/11 lambs. Three lambs achieved ROSC with the first precompression ETT dose of epinephrine, 1 lamb achieved ROSC following 2 doses of ETT epinephrine, and 6 of the remaining 7 lambs achieved ROSC following intravenous epinephrine. One lamb that received 2 doses of ETT followed by intravenous epinephrine developed a ventricular tachycardia during resuscitation, which was initially treated with calcium gluconate without success. A rhythm strip appeared to show torsade de pointes (Figure 2) and magnesium sulfate and lidocaine were administered in an attempt to convert to normal sinus rhythm. The lamb converted to sinus rhythm and achieved ROSC at 42 minutes and sustained normal hemodynamic parameters until sacrifice. As an extreme, atypical clinical course, this lamb was counted as a successful ROSC but the time to ROSC was omitted from the average mean.

**Figure 3:**
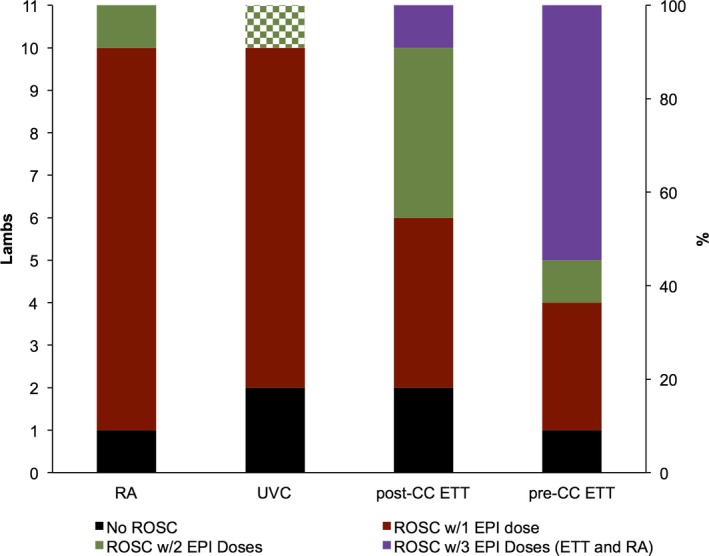
Animals achieving ROSC with varied epinephrine route by group. In the UVC group, the checkered bar represents 4 UVC epinephrine doses. The purple bars represent the lambs that received 2 ETT epinephrine doses followed by 1 dose of RA epinephrine prior to achieving ROSC. CC indicates chest compression; EPI, epinephrine; ETT, endotracheal tube; RA, right atrium; ROSC, return of spontaneous circulation; UVC, umbilical venous catheter.

The incidence of ROSC by group is shown in Figure 3.

### Hemodynamic Parameters

**Figure 4:**
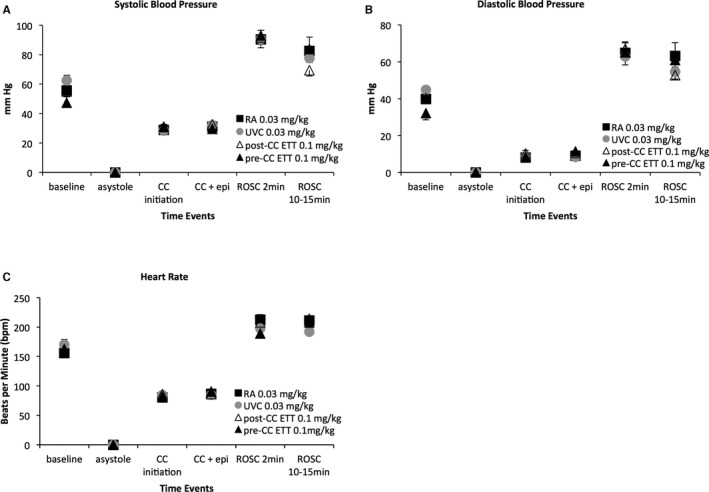
Systolic (A) and diastolic (B) blood pressures, and heart rate (C) are similar among groups. Epinephrine did not significantly increase systolic (A) or diastolic (B) pressures during chest compressions. The chest compression rate was maintained around 90 per minute (C). In the lambs that achieved ROSC, there was a significant increase in systolic (A) and diastolic (B) blood pressures that started to improve by the end of the study. Heart rate stayed high following ROSC (C). Data are mean±SEM. CC indicates chest compression; EPI, epinephrine; ETT, endotracheal tube; RA, right atrium; ROSC, return of spontaneous circulation; UVC, umbilical venous catheter.

**Figure 5:**
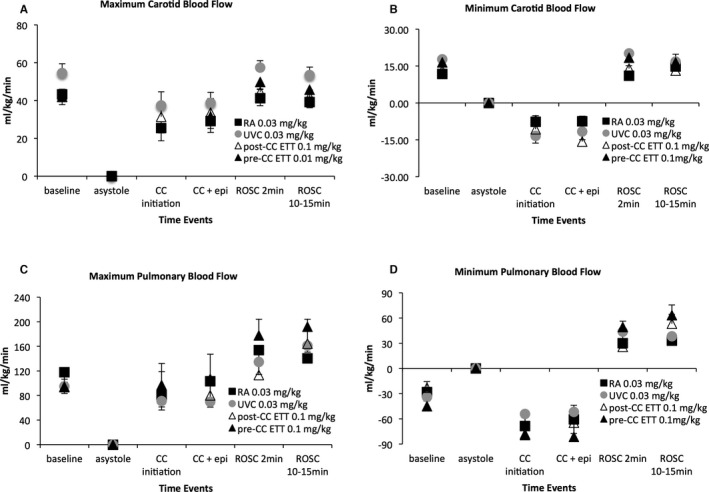
Maximum (A) and minimum (B) left carotid blood flows, and maximum (C) and minimum (D) left pulmonary blood flows are similar among groups. Epinephrine did not significantly increase blood flows during chest compression. Minimum left carotid (B) and minimum left pulmonary (D) blood flows are retrograde during the relaxation (“diastolic”) phase of chest compressions. Minimum pulmonary blood flows (D) are also retrograde in the fetus at baseline because of the high pulmonary vascular resistance in utero. Data are mean±SEM. CC indicates chest compression; ETT, endotracheal tube; RA, right atrium; ROSC, return of spontaneous circulation; UVC, umbilical venous catheter.

There were no significant differences at any time in the systolic and diastolic blood pressures, left carotid and left pulmonary blood flows, and the heart rate among the groups (Figures 4 and 5). At the onset of chest compressions, the systolic blood pressures ranged from 25 to 28 mm Hg, which increased to 29 to 33 mm Hg following epinephrine. The diastolic blood pressures followed a similar pattern with pressures of ≈6 to 8 mm Hg at the beginning of chest compressions that increased to 9 to 13 mm Hg following epinephrine.

Following ROSC, there was a significant increase in systolic and diastolic blood pressures (Figure 4A and 4B). However, blood pressures started normalizing by the end of the study period (69–83/52–60 mm Hg), similar to historical nonasphyxiated control term lambs during the first 10 minutes of postnatal age (65–108/50–62 mm Hg). The chest compression rate was maintained between 85 and 90 compressions per minute during resuscitation. The heart rates quickly increased upon ROSC and tachycardia persisted for the duration of the study (Figure 4C). When analyzing the lambs that achieved ROSC following only ETT epinephrine (n=12), blood pressures and heart rate after ROSC also rose to similar values compared to the intravenous groups.

**Figure 6:**
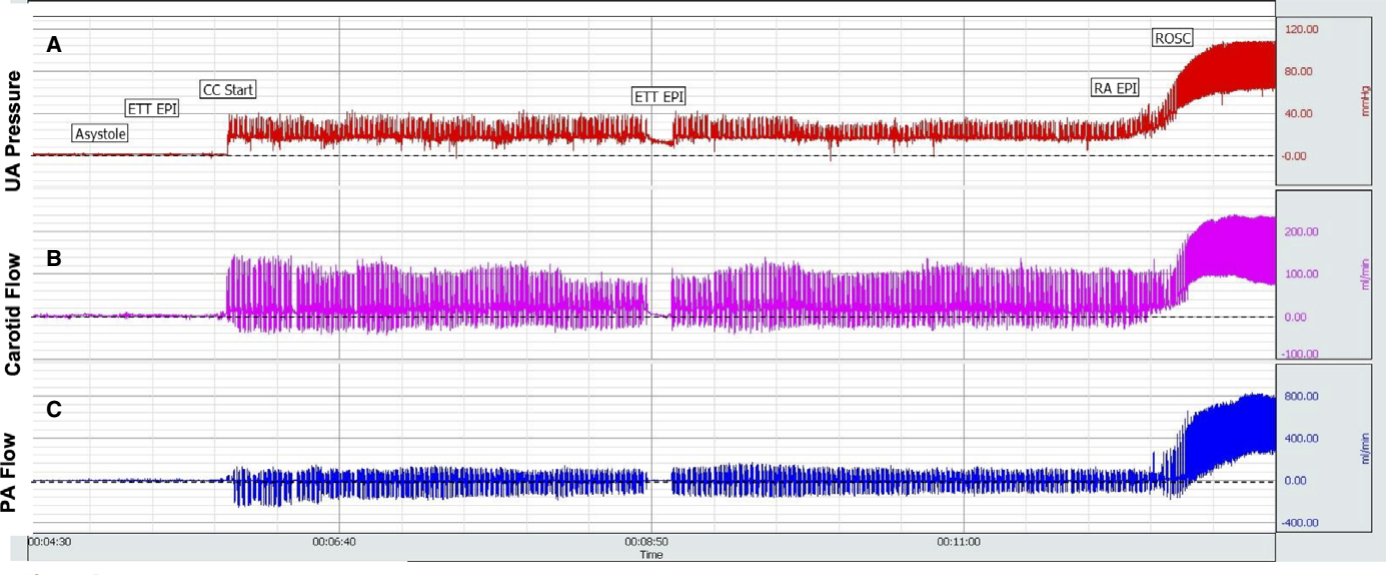
Snapshot of Biopac showing umbilical arterial pressure (A), left carotid blood flow (B), and left pulmonary arterial blood flow (C) from asystole through resuscitation and ROSC. Dashed line represents value “0.” Blood flows below dashed lines represent retrograde flow. With ROSC and cessation of chest compressions, blood flow is exclusively anterograde. CC indicates chest compressions; ETT, endotracheal tube; PA, pulmonary artery; RA, right atrium; ROSC, return of spontaneous circulation; UA, umbilical artery.

The maximum carotid and pulmonary blood flows increased following epinephrine administration but did not achieve statistical significance as compared to the period prior to epinephrine administration (Figure 5A and 5B). An interesting observation during resuscitation was the negative minimum carotid and pulmonary blood flow values, where reversal of flow occurred during the relaxation (“diastolic”) phase of chest compressions (Figure 5B and 5D). Following ROSC, these values became positive, demonstrating forward flow in the carotid and pulmonary arteries during the systolic as well as diastolic phases of the cardiac cycle (Figure 6).

### Plasma Epinephrine Concentrations

**Figure 7:**
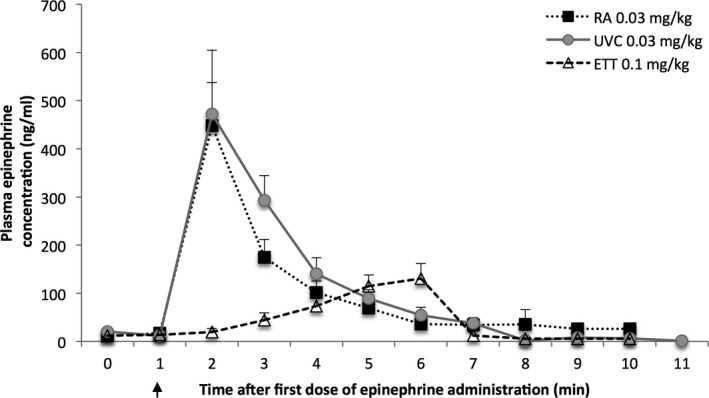
Plasma epinephrine concentrations from lambs that received 1 dose of epinephrine. The precompression ETT and postcompression ETT groups were combined in this graph. Arrow indicates time of epinephrine administration. Data are mean±SEM. ETT indicates endotracheal tube; RA, right atrium; UVC, umbilical venous catheter.

Baseline and arrest plasma epinephrine concentrations were similar, ranging from 3 to 20 ng/mL. Results from lambs that received only 1 dose of epinephrine (ETT, UVC, or RA) are shown in Figure 7. Intravenous injection of epinephrine (0.03 mg/kg) through the jugular catheter into the right atrium or into the umbilical vein through a low‐lying UVC resulted in similar peak plasma concentrations (470±250 versus 450±190 ng/mL) within 1 minute of epinephrine administration. Instillation of ETT epinephrine (0.1 mg/kg) resulted in significantly lower (130±60 ng/mL) and delayed (4–5 minutes following epinephrine administration) peak concentrations; *P*<0.05.

**Figure 8:**
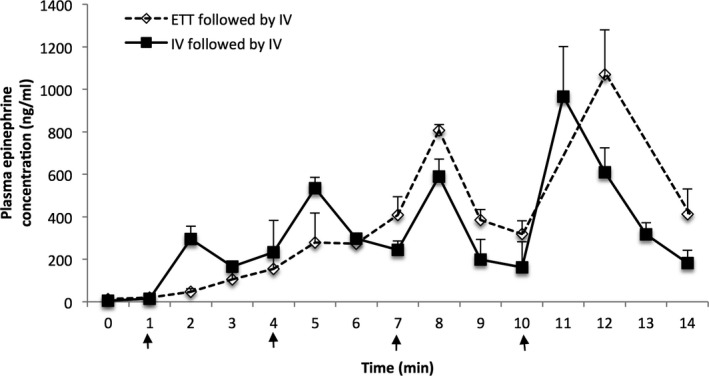
Plasma epinephrine concentrations from lambs that received multiple doses of epinephrine. All lambs in the *ETT followed by IV* group required at least 1 dose of intravenous epinephrine. Arrows indicate epinephrine administration. In the *IV followed by IV* group, all doses were IV at 0.03 mg/kg. In the *ETT followed by IV* group, epinephrine for the first 2 doses was 0.1 mg/kg by ETT, followed by IV epi at 0.03 mg/kg. Data are mean±SEM. ETT indicates endotracheal tube.

**Figure 9:**
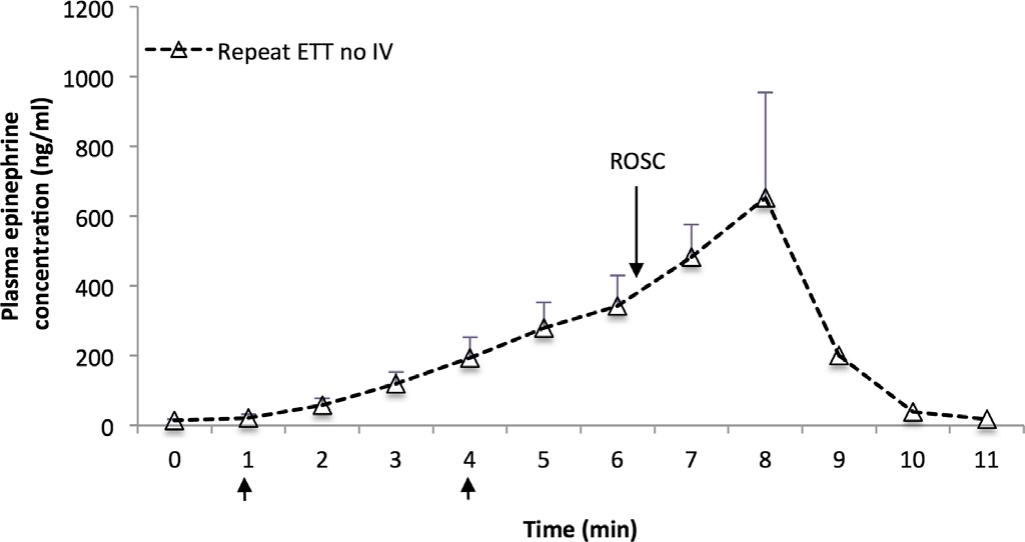
Plasma epinephrine concentrations in lambs that achieved ROSC following 2 doses of tracheal epinephrine without any intravenous epinephrine. The peak value at 8 minutes (ie, 4 minutes after the last ETT dose) occurred following ROSC. Arrows indicate ETT epinephrine at 0.1 mg/kg. Data are mean±SEM. ETT indicates endotracheal tube; ROSC, return of spontaneous circulation.

Results from the lambs that received multiple doses of epinephrine are presented in Figure 8. When analyzing the data of the lambs randomized to the ETT epinephrine groups that also received IV epinephrine, there was a slow rise in the plasma epinephrine concentrations following the ETT epinephrine doses (peak of ≈350 ng/mL at 6 minutes), whereas a sharp rise in plasma concentration was observed following the intravenous dose (peak of ≈800 ng/mL). In the lambs that exclusively received intravenous epinephrine, there was a steady cumulative rise in plasma concentration after each epinephrine injection to peak values exceeding 1000 ng/mL in the animals that received a total of 4 doses. Interestingly, there was also a continued increase in the plasma epinephrine concentrations following ROSC in the lambs that received only ETT epinephrine (Figure 9). Multiple epinephrine doses were associated with tachyarrhythmias in 3 lambs (1/11 lambs in the UVC group, and 2/22 in the ETT groups following an IV dose).

### Arterial Blood Gas Analysis

There were no significant differences in the PaO_2_, PaCO_2_, lactate, and pH among the groups throughout the resuscitation period (adequate ventilation was achieved by monitoring tidal volumes). In the lambs that were successfully resuscitated (excluding the lamb with an arrhythmia that achieved ROSC after a prolonged resuscitation; n=37), the arterial blood gas samples collected at the time of arrest, 1 minute preceding ROSC, at ROSC, 1 minute following ROSC, and 10 minutes following ROSC are shown in Table 2. The hemoglobin concentration remained constant with an average hemoglobin concentration of 12.1±1.4 g/dL by the end of the study. Thus, the lambs achieved ROSC with a mean arterial oxygen tension of 25 mm Hg (fetal hemoglobin saturation of 65%) equivalent to an oxygen content of 10.7 mL/dL. With low mean carotid blood flows during chest compressions, mean oxygen delivery from the left carotid artery was only 4.3±1.2 mL O_2_/kg per minute (compared to 25±6.3 mL O_2_/kg per minute during fetal baseline and 40±9.1 mL O_2_/kg per minute 10 minutes after ROSC). Also, very high PaO_2_ values (>200 mm Hg) were seen within the first few minutes following ROSC despite gradually weaning the FIO_2_ once ROSC was confirmed. The PaO_2_ normalized within the first 10 minutes when all lambs had been weaned to an FIO_2_ of 0.21 (Table 2).

**Table 2:** Arterial Blood Gas Analysis in the 4 Study Groups

	Group 1 RA	Group 2 UVC	Group 3 Postcompression ETT	Group 4 Precompression ETT
At arrest	N=11	N=11	N=11	N=11
pH	6.85±0.07	6.80±0.09	6.85±0.06	6.85±0.04
PaCO_2_, mm Hg	130±27	146±25	139±17	132±17
PaO_2_, mm Hg	5.5±3.5	6.0±4.5	6.0±4.5	5.8±3.5
Lactate, mmol/L	12.1±5.4	14.6±6.7	13.9±4.9	14.8±5.1
1 minute before ROSC	N=10	N=9	N=9	N=9
pH	6.88±0.15	6.80±0.16	6.85±0.06	6.83±0.07
PaCO_2_, mm Hg	117±27	124±45	106±15	118±27
PaO_2_, mm Hg	24±9.1	25±6.9	25±6.6	24±6.2
Lactate, mmol/L	13.6±3.5	13.5±5.9	15.3±4.5	16±4.8
At ROSC	N=10	N=9	N=9	N=9
pH	6.82±0.06	6.79±0.16	6.86±0.06	6.81±0.07
PaCO_2_, mm Hg	116±29	115±47	97±14	115±39
PaO_2_, mm Hg	119±127	161±209	90±106	157±173
Lactate, mmol/L	14.4±2.4	14.5±5.1	14.8±4.5	16.5±4.9
1 minute after ROSC	N=10	N=9	N=9	N=9
pH	6.85±0.09	6.79±0.11	6.90±0.10	6.91±0.11
PaCO_2_, mm Hg	100±41	105±29	94±25	101±22
PaO_2_, mm Hg	261±148	221±204	208±136	224±173
Lactate, mmol/L	13.5±1.4	15.4±3.1	14.7±4.1	15.5±5.3
10 minute after ROSC (FIO_2_ 0.21)	N=10	N=9	N=9	N=9
pH	7.07±0.15	7.00±0.99	7.09±0.13	7.01±0.12
PaCO_2_, mm Hg	56±28	66±19	50±19	54±14
PaO_2_, mm Hg	81±34	63±11	71±17	74±28
Lactate, mmol/L	14.4±5.5	14.3±5.4	12.4±3.8	15.3±5.7

N represents the number of lambs. Data are mean±SD. ETT indicates endotracheal tube; RA, right atrium; ROSC, return of spontaneous circulation; UVC, umbilical venous catheter.

## Discussion

Epinephrine has been used in cardiopulmonary resuscitation since the early 1960s.^12^ However, because of the infrequent use of medications during neonatal resuscitation, there is a lack of rigorous scientific evidence for the effects of epinephrine use in the neonatal population. Therefore, the appropriate dosing, timing, order, and route of epinephrine administration remain controversial.^3^ This study is the first attempt to evaluate the efficacy, pharmacokinetics, and safety profile of ETT and low UVC epinephrine in a newborn asphyxial arrest model. This model includes 5 minutes of asystole following cord occlusion resembling severe asphyxia with cardiac arrest, which typically requires PPV, chest compressions, and epinephrine to achieve ROSC.

The use of high‐dose IV epinephrine (0.2 mg/kg) in a pediatric swine cardiac arrest model has been associated with severe tachycardia, hypertension, and higher mortality in the immediate postresuscitation period.^13^ In neonatal lambs, the use of high‐dose IV epinephrine (0.1 mg/kg) results in reduced stroke volume and cardiac output.^14^ In addition, there is no evidence of improved clinical outcomes to support the use of high‐dose intravenous epinephrine in adults.^15^, ^16^ The findings from the current study establish the efficacy of epinephrine administered into the RA at a dose of 0.03 mg/kg followed by a 0.5 mL/kg normal saline flush. Optimal plasma concentrations similar to that described in other animal models were achieved with a high incidence of ROSC (10/11 lambs) at 2.4±1.1 minutes after the onset of resuscitation (or 84±65 s after intravenous epinephrine administration).^6^ Seven additional lambs in the ETT groups achieved ROSC following IV RA epinephrine. Time to ROSC following the RA epinephrine dose in those 7 lambs was 82±33 s.

Administration of epinephrine at the same dose (0.03 mg/kg followed by 0.5 mL/kg of flush) into the umbilical vein through a low‐lying UVC resulted in similar peak plasma concentrations and similar rates of ROSC (9/11 lambs). Time to ROSC was prolonged as compared to RA epinephrine (3.1±2.7 minutes after onset of resuscitation or 126±122 s after epinephrine administration) although this difference was not statistically significant.

The high frequency of initial use of ET epinephrine clinically (while attempting to establish UVC access) makes it critical that the recommended dose be as effective as possible. Studies in newborn piglets (2–4 days old) observed no increase in plasma epinephrine concentrations after the recommended IV epinephrine dose (0.01 mg/kg) is administered via ETT.^6^ Another piglet study has shown that higher‐dose ETT epinephrine (0.07 mg/kg) is associated with an 89% success rate in achieving ROSC.^17^ Therefore, when administering endotracheal epinephrine, a higher dose is likely required to compensate for the lung liquid dilution as well as to overcome the diffusion barrier at the level of the alveolar capillaries. In our study, we elected to administer the higher range of endotracheal epinephrine at a dose of 0.1 mg/kg as currently recommended by the Neonatal Resuscitation Program.^18^ Out of the 22 lambs that received ETT epinephrine, only 7 (32%) achieved ROSC following the first dose of epinephrine. Another 5 lambs were successfully resuscitated following a second dose of ETT epinephrine (total ETT epinephrine ROSC success of 12/22=55%). However, the epinephrine concentrations achieved were significantly lower and peak concentrations were markedly delayed compared to RA or UVC administration of epinephrine (Figure 7). Another concern with endotracheal administration was the high plasma epinephrine concentrations achieved after establishment of ROSC (Figure 8). We speculate that improved pulmonary blood flow and slow absorption of epinephrine from the fluid‐filled lungs resulted in higher plasma concentrations. Though we did not observe any hemodynamic compromise in the immediate recovery phase (lambs were monitored for 30 minutes from onset of resuscitation), previous studies have shown that ETT epinephrine can worsen the hemodynamics by reducing systemic vascular resistance due to greater β‐ versus α‐adrenergic effects.^19^, ^20^ Therefore, the depot effect and the delayed absorption following ETT epinephrine, especially if IV epinephrine has also been administered, may potentiate increased adverse effects after ROSC because of the high plasma epinephrine concentrations. Following IV epinephrine, a total of 19/22 lambs in the endotracheal groups achieved ROSC, which is comparable to the IV groups. Further studies evaluating the safety profile of multiple ETT epinephrine doses (at 0.1 mg/kg) are necessary. Contrary to our hypothesis, precompression administration of ETT epinephrine was not effective in restoring ROSC (4/11 lambs). Our assumption that instilling epinephrine into the ETT while providing PPV to distribute the medicine more homogeneously into the fluid‐filled lungs prior to starting chest compressions to enhance/hasten the effects of epinephrine was incorrect. In the absence of chest compressions and pulmonary blood flow, epinephrine absorption from the lungs is not adequate.

Three lambs developed tachyarrhythmias: 1 following administration of multiple UVC epinephrine doses during resuscitation and could not be converted, another ≈2 minutes after ROSC was achieved following RA epinephrine plus 2 earlier doses of ETT epinephrine (also could not be converted), and, finally, 1 developed arrhythmias during resuscitation and ultimately converted to sinus rhythm following administration of calcium gluconate, magnesium sulfate, and lidocaine. Tachyarrhythmias have been previously reported in a piglet newborn model comparing the efficacy of vasopressin with epinephrine.^21^ Neonatal healthcare providers should be aware that severely compromised neonates are at risk for development of arrhythmias if multiple doses of epinephrine are administered. The 2015 Neonatal Resuscitation Program guidelines recommendation for ECG monitoring in the delivery room may lead to increased identification of arrhythmias.^22^

Hemodynamic effects of epinephrine administration in a newborn model with patent fetal shunts (ductus arteriosus, patent foramen ovale, and ductus venosus) have been better characterized by our study. In this neonatal asphyxial cardiac arrest model with transitioning fetal circulation, we observed significant retrograde pulmonary and carotid blood flow during the relaxation (“diastolic”) phase of chest compressions, the latter corroborating findings by Sobotka et al.^10^ Furthermore, contrary to studies in adult and pediatric models, epinephrine did not significantly elevate blood pressure or coronary perfusion pressure.^10^, ^23^, ^24^, ^25^, ^26^ Coronary perfusion pressure acts as a surrogate for myocardial blood flow and in the intrinsically beating heart, myocardial blood flow is at its greatest during diastole when the heart muscles relax. However, in the arrested neonatal heart with fetal shunts, it is not known whether myocardial blood flow is greatest during compression (systole) or relaxation (diastole). Our data suggest that systolic blood pressures may be more predictive of ROSC as the lambs in this study achieved ROSC with low mean diastolic blood pressures (≈10 mm Hg).

We speculate that the rapid achievement of ROSC was associated with the higher and more rapid plasma epinephrine concentration achieved with intravenous epinephrine, mediated by epinephrine's β‐adrenergic effects on the myocytes, as well as on the sinoatrial and atrioventricular nodes, leading to improved impulse generation, conduction velocity, and contractility during chest compressions.^27^ The diminished response in elevating the blood pressures suggests that the α‐adrenergic effects of epinephrine in this severely asphyxiated acidotic model may be blunted during resuscitation.

Finally, the significant rise in PaO_2_ following ROSC when ventilating in 100% oxygen raises concern of the potential deleterious effects of excess oxidative stress and risk of free radical generation with risk of potentiating long‐term neurologic deficits.^11^, ^28^, ^29^, ^30^ We therefore suggest decreasing the FIO_2_ to 0.21 immediately following ROSC and, thereafter, adjust the FIO_2_ accordingly to maintain saturations in the goal ranges.^31^

We acknowledge several limitations to this study. A flow probe was not placed around the ductus arteriosus, limiting our interpretation and understanding of the hemodynamics of chest compression during resuscitation in a model with transitioning circulation. Instrumentation of the fetal lambs was performed just prior to our study and may have induced stress. In addition, although we sutured the thoracotomy in layers, we may have altered mechanical properties of the thoracic cage during chest compressions. Finally, we cannot be certain that some lambs may achieve ROSC if the time to first epinephrine administration is extended.

## Conclusions

This is the first reported study in the literature investigating the effects and pharmacokinetics of epinephrine administration at different sites (RA, low UVC, and ETT route) in a neonatal asphyxial cardiac arrest model with transitioning fetal circulation and fluid‐filled lungs. RA and low UVC epinephrine administration achieve significantly higher and quicker peak plasma epinephrine concentrations with a high ROSC success rate (81–91%), whereas the systemic absorption of ETT epinephrine through liquid‐filled lungs is low and delayed. Future studies evaluating a single higher dose of epinephrine (0.2 mg/kg) are warranted.

## Sources of Funding

This work was supported by the American Association of Pediatrics Neonatal Resuscitation Program Young Investigator Award (Vali), Canadian Paediatric Society (Lakshminrusimha), and NIH HD072929 (Lakshminrusimha). Dr Jusko is supported by National Institutes of Health Grants GM 24211 and NICHHD HD071594.

## Disclosures

None.
